# MIStore: a Blockchain-Based Medical Insurance Storage System

**DOI:** 10.1007/s10916-018-0996-4

**Published:** 2018-07-02

**Authors:** Lijing Zhou, Licheng Wang, Yiru Sun

**Affiliations:** State Key Laboratory of Networking and Switching Technology, Beijing University of Posts and Telecommunications, Beijing, 100876 China

**Keywords:** Medical insurance, Blockchain, Secret sharing, Multi-parties computing

## Abstract

Currently, blockchain technology, which is decentralized and may provide tamper-resistance to recorded data, is experiencing exponential growth in industry and research. In this paper, we propose the MIStore, a blockchain-based medical insurance storage system. Due to blockchain’s the property of tamper-resistance, MIStore may provide a high-credibility to users. In a basic instance of the system, there are a hospital, patient, insurance company and *n* servers. Specifically, the hospital performs a (*t*, *n*)-threshold MIStore protocol among the *n* servers. For the protocol, any node of the blockchain may join the protocol to be a server if the node and the hospital wish. Patient’s spending data is stored by the hospital in the blockchain and is protected by the *n* servers. Any *t* servers may help the insurance company to obtain a sum of a part of the patient’s spending data, which servers can perform homomorphic computations on. However, the *n* servers cannot learn anything from the patient’s spending data, which recorded in the blockchain, forever as long as more than *n* − *t* servers are honest. Besides, because most of verifications are performed by record-nodes and all related data is stored at the blockchain, thus the insurance company, servers and the hospital only need small memory and CPU. Finally, we deploy the MIStore on the Ethererum blockchain and give the corresponding performance evaluation.

## Introduction

Bitcoin, proposed in 2009 by Satoshi Nakamoto [1], is the first decentralized cryptocurrency which maintains a public transaction ledger, called blockchain, in a distributed manner without the central authority. The core technological innovation of Bitcoin is Nakamoto consensus which provides a high-probability guarantee that an adversary cannot alter a transaction once this transaction is sufficiently deep in the blockchain, assuming honest nodes control the majority of computational resources in the system. The Nakamoto blockchain works in a permissionless model, where any node can freely join and leave the protocol, and there is no a-priori knowledge of the set of consensus nodes. Alternative cryptocurrencies called altercoins (e.g., Litecoin [2], Ripple [3] and Ethereum [4]) have achieved enormous success. Several consensuses to manage blockchain-based ledgers have been proposed: proof-of-work [5], proof-of-stake [6, 7], proof-of-space [8], proof-of-activity [9], proof- of-human-work [10], practical Byzatine fault-tolerance [11], or some combinations [12–14]. Especially, most existing cryptocurrencies, including Bitcoin, adopt proof-of-work.

Blockchain is a tamper-resistant timestamp ledger of blocks that is utilized to share and store data in a distributed manner. The stored data may be payment records (e.g., Bitcoin, Litecoin), contract (e.g., Ethererum) or personal data. Currently, blockchain has attracted enormous attention from academics and practitioners (e.g., computer science, finance and law) due to its signal properties containing distributed structre, security, privacy and immutability [17]. In blockchain, users can generate a arbitrary number of public keys that effectively prevents them from being tracked, and this ensure users’ privacy. Recently, blockchain has been widely utilized in non-monetary applications including but not limited to: securing robotic swarms [19] and verifying proof of location [20]. Moreover, blockchain can use cryptography technologies [31–33] to improve it security, privacy and functionality.

Recently, blockchain-based medical system is a hot topic. Yue et al. [18] proposed a APP of sharing healthcare data, where patients control, send and own their data easily. Moreover, Qi et al. [30] proposed the MeDShare, a system that can address the problem of medical data sharing among medical big data servers in a trust-less environment. Besides, Ekblaw et al. [29] presented the MedRec, decentralized record management system to resolve electronic health records by using blockchain. In the researches, they did not provide the function of homomorphic computing for data recorded at the blockchain, and they just utilized the blockchain as a storage tool. Therefore, in the systems, a node cannot help others to process encrypted data.

In an ideal and basic medical insurance business, there are a hospital, a patient and an insurance company. The insurance company can to know a sum of the patient’s specified spending records, however the company cannot learn the details of the spending records. Furthermore, servers can help the insurance company to process a patient’s spending records without learning anything about the spending records. Otherwise, it will result in a risk of information leakage. Moreover, once the insurance company attempt to know the patient’s sum of spending records, the insurance company can get his desired result without any help of hospital and patient. Finally, the most important point is that all data must be verifiable and tamper-resistant. Otherwise, the system could not be credible.

To address the problems, in the present paper, we propose the MIStore, a blockchain-based medical insurance storage system. Features of MIStore can be summarized as follows:

- **Decentralization.** There is no the third party authorities to provide any authentication. Moreover, any node may become some hospital’s server if the node and the hospital wish. Besides, data is stored at the blockchain, rather than cloud servers.
- **Secure data storage.** On the one hand, every transaction’s publicly verifiable data must be verified by all record-nodes before the transaction is included in the blockchain. On the other hand, we suggest that MIStore adopts the Practical Byzantine Fault-tolerance (PBFT) to be the consensus scheme of the blockchain, and all related data is stored at the blockchain. Due to PBFT’s property of tamper-resistance, data, which has been included by record-nodes in the blockchain, cannot be modified or deleted by anyone. Due to the above two points, it provides high credibility to all users. Therefore, once a transaction has been included in the blockchain, all its publicly verifiable data is credible.
- **Threshold.** For instance, a hospital performs a (*t*, *n*)-threshold MIStore protocol among a patient, an insurance company and *n* servers. Firstly, the hospital store confidential data in the blockchain. Secondly, the servers cannot learn anything from the data if more than *n* − *t* servers are honest. Thirdly, after the insurance company sends a query to the blockchain, if he can collect *t* correct responses to the query, then he can obtain his desired result. Finally, anyone (including the insurance company) cannot learn anything with less than *t* responses.
- **Verifiable.** Key data stored at the blockchain is verifiable. Specifically,
  - Anyone can verify whether the verification key is valid.
  - Any server can verify whether his core-share is correctly computed by the corresponding hospital.
  - The insurance company can verify whether responses are correctly computed by corresponding servers, respectively.
  - Patient may verify whether his spending data is correctly processed by corresponding hospital.
- **Efficient verification.** Key data is recorded in the transaction’s payload, and most of key data is publicly verifiable. Therefore, record-nodes can help other nodes to verify payloads’ data before the transactions are recorded in the blockchain. Consequently, once a transaction has been recorded in the blockchain, the transaction’s publicly verifiable data is credible. After that, the transaction’s receiver needs not to perform the verifications performed by record-nodes. Moreover, the receiver just needs to perform very little verification that can be performed only by him. In this way, it significantly reduces users’ verifying computations, and receivers just perform some simple and few computations, rather than complex and massive computations.
- **Efficient homomorphic computation.** According to insurance company’s query, servers can perform homomorphic multiplications and additions on their shares, and then generate responses. Moreover, the homomorphic computations calculated by servers are efficient additions and multiplications of finite field.

### Organization

In “Background”, background is introduced. In “System setting and model”, we show the system setting and model. In “An overview of MIStore”, an overview of MIStore is given. We introduce construction of the MIStore system in “MIStore”. In “Performance evaluation”, a performance evaluation is given. Finally, a short conclusion is given in “Conclusion”.

## Background

Bitcoin [1] is a decentralized payment scheme in which every participant maintains its own local copy of the whole transaction history, “chain” of “blocks” called blockchain. Blockchain is maintained by anonymous record-nodes, called miners, via executing a consensus scheme that extends the blockchain. The record-nodes are connected by a reliable peer-to-peer network. Bitcoin consistency relies on the idea of computational puzzles—a.k.a. moderately proof-of-work put forth by Dwork and Naor [16]. In Bitcoin, payers broadcast transactions and miners collect transactions into their local blocks. A block contains two parts: block-body and block-header. Specifically, the block-body contains the transactions. The block-header contains the hash value of previous block, the current Unix time, target value, a nonce and a merkle root of transactions. In Bitcoin consensus, a block to be valid if the cryptographic hash of its header must be smaller than a target value. Moreover, if some miner finds a solution of the cryptographic puzzle, then he immediately broadcasted his block including the solution to others. After that, upon verifying the block, others will receive and add this block as a new one in its local blockchain and then continue the mining process on its updated blockchain. The creator of the block is rewarded with bitcoins (coins in Bitcoin system) via the coinbase transaction which is the first transaction in the block-body. Consequently, bitcoins are created and distributed among miners. Moreover, this creator is also rewarded by transactions fees for all transactions included in the block. Besides, Bitcoin assumes that a majority of computational power is controlled by honest players.

Smart contract is proposed by Ethereum [15] that is similar as Bitcoin. Smart contracts represent the implementation of a contractual agreement, whose legal provisions have been formalized into source code. Contracting parties can structure their relationships efficiently, in a self-executing method and without the ambiguity of words. Reliance on source code enables willing parties to simulate the agreement’s performance before execution and model contractual performance. Moreover, smart contracts introduce new relationships that are both automatically enforced and defined by code, but that are not linked to any underlying contractual rights or obligations. In the present paper, before hospital and servers work, they should mortgage coins in smart contracts, respectively. Besides, if someone does not work honestly, then anyone can input the corresponding evidences to obtain a part of the “wrongdoer”’s guarantee deposit.

In the paper, the security of Shamir’s (*t*, *n*)-secret sharing (SSS) [21] is the security base of our system. We extend SSS to obtain a threshold secure multi-parties computing protocol that will be described in the A. Besides, we use elliptic curve [22, 23] point multiplication to generate commitments of core data. Then we utilize bilinear map (pairing computations) [25] to verify the correctness of the committed core data.

Figures presented in the paper are created by using Visio.

## System setting and model

### Blockchain network and cryptographic keys

MIStore is comprised of record-nodes and light-nodes. Specifically, all record-nodes are connected by a reliable peer-to-peer network, and each light-node connects with a certain number of record-nodes. Record-nodes are responsible to maintain the blockchain via Practical Byzantine Fault-tolerance (PBFT) consensus and store the entire blockchain list. Specifically, time is divided in to epoches. In an epoch, record-nodes collect and verify transactions sent to the blockchain network, and they record valid transactions in their local blocks. By performing PBFT, some record-node’s block become the valid block of the epoch. After that, all record-nodes join in the next epoch to build the next block. While, light-nodes do not store the entire blockchain, and they store all block-headers.

Moreover, in the system, there is no trusted public key infrastructure. It means that any node can generate a arbitrary number of key-pairs by itself. In a blockchain system, all users communicate with each other via transactions of blockchain, and they only trust messages presented at blockchain. Additionally, each record-node can poll a random oracle [24] as a random bit source. Besides, by mortgaging a certain amount of coins with an address, a light-node can become a hospital or insurance company with the address.

A node is honest if it follows all protocol instructions and is perfectly capable of sending and receiving information. Furthermore, a node is malicious if it can deviate arbitrarily from protocol instructions. Finally, in a blockchain system, all users communicate with each other via transactions of blockchain, and they only trust messages presented at blockchain.

In the implementation of MIStore, we utilize ECDSA [27] to be the signature schceme Sig(⋅), ECIES [28] to be the encryption scheme Enc(⋅) and SHA-256 [1] to be the hash function H(⋅).

### Assumptions

According to Practical Byzantine Fault-tolerance [11] consensus scheme, we assume that $\frac {2}{3}$ of record-nodes are honest in the system. Therefore, the blockchain of the system does not fork. In other words, once a transaction has appeared in the blockchain, then the transaction cannot be modified or deleted by anyone. Moreover, we assume that digital signature Sig(⋅), encryption scheme Enc(⋅) and hash function H(⋅) used in MIStore are ideal such that no one can violate Sig(⋅), Enc(⋅) and H(⋅). Finally, we assume that hospital and servers are partially trusted. Therefore, data sent by them should be verified.

#### The basic instance

In the paper, we mainly introduce the *basic instance* that contains a patient, a hospital and insurance company. For the patient, a more complex instance can be combined by the basic instance. Moreover, we assume that medical payments recorded in the blockchain belong to the range of the corresponding medical insurance.

## An overview of MIStore

In this paper, we propose a blockchain-based medical insurance storage system, called MIStore. The system may help an insurance company to obtain the sum of patient’s medical medical spending records. Moreover, the medical spending data recorded at the blockchain are always confidential to servers as long as a certain number of servers are honest. In this system, there are four parties that are patients, hospitals, servers and insurance companies. All related data is recorded at the blockchain. Due to the property of tamper-resistance of blockchain, all users may trust data recorded at the blockchain.

**Fig. 1:**
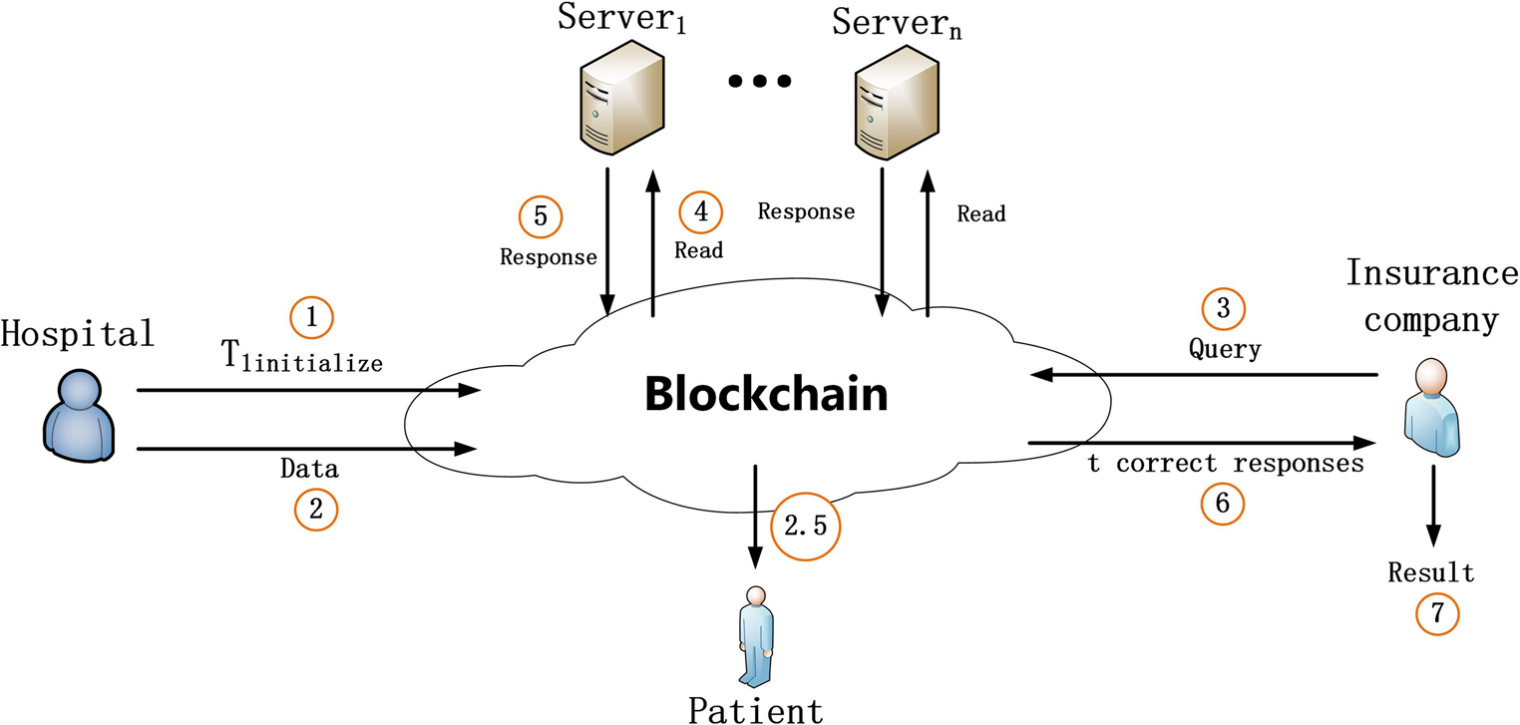
An overview of MIStore. Step 1: Hospital sends a initialize-transaction to blockchain networl. Step 2: Hospital sends record-transactions to blockchain network. Step 2.5: The patient can verify whether his spending records are correctly computed by hospital. Step 3: Insurance company sends a query-transaction to query some result. Step 4: Servers read the query-transaction and related record-transactions from blockchain. Step 5: After locally computing, servers generate their responses and then send respond-transactions to blockchain network. Step 6: Insurance company collects respond-transactions and obtains *t* correct responses. Step 7: Insurance company recovers the result with the *t* correct responses

To introduce MIStore’s working process, we take the basic instance as a example, which contains a hospital *H*, a patient *P*, an insurance company *I* and *n* servers *Sr*_1_, *Sr*_2_, ⋯ *Sr*_*n*_. Specifically, *H* performs a (*t*, *n*)-threshold MIStore protocol among the *n* servers by sending an initialize-transaction to the blockchain network. After that, *H* may send *P*’s confidential medical spending data to the blockchain network by sending record-transactions. At some later time, if *I* wants to know the sum of some *P*’s spending records, then *I* may send a query-transaction to the blockchain network. After that, active servers will generate and send responses to the blockchain network by sending respond-transactions. Finally, if *I* collects at least *t* correct responses, then he can recover the real result. However, it must be pointed that anyone (including *I*) cannot learn anything about the correct result with less than *t* responses. The protocol is secure as long as more than *n* − *t* servers are honest. An overview of MIStore is shown in Fig. 1.

In the MIStore, most data is verifiable. For instance, anyone can verify the validation of the initialize-transaction sent by the hospital, a server may verify the correctness of his core share sent by the hospital, an insurance company can verify whether responses are correctly computed by corresponding servers, and a patient can verify whether his spending data is correctly processed by the corresponding hospital. Moreover, because MIStore is decentralized, so there is no centralized node to punish the “bumblers”. To punish the bumblers’ mistake, we adopt the smart contract. Specifically, before hospitals and servers perform a MIStore protocol, they should a certain amount of mortgage coins in smart contracts, respectively. If someone of them publishes some invalid data in the blockchain, then anyone can input the evidences in the corresponding smart contract to obtain a part of the bumbler’s guarantee deposit.

## MIStore

In this section, we introduce how MIStore works. We will describe *transaction and block* used in the system at first.

### Transaction and block

In MIServer, a transaction contains two parts that are *transaction header* and *payload*. Transaction header and payload are shown in Table 1.

**Table 1:** Format of transaction

Transaction header	
Hash	The transaction’s hash value
Block number	Block containing the transaction
Order	The transaction’s number in the block
Timestamp	Creation time of the transaction
Sender	Sender’s ID
Receiver	Receiver’s ID
Signature	Sig{the transaction’s hash value}
Payload: Data	
data_1_, data_2_, ⋯, data_*n*_	

Moreover, the payload might contain secret or public data that may be used in verifications or computations. In the system, according to payload, transactions can be divided into four types. They are initialize-transaction, record-transaction, query-transaction and respond-transaction, and they can be described by *T*_*initialize*_, *T*_*record*_, *T*_*query*_ and *T*_*respond*_ as follows: 
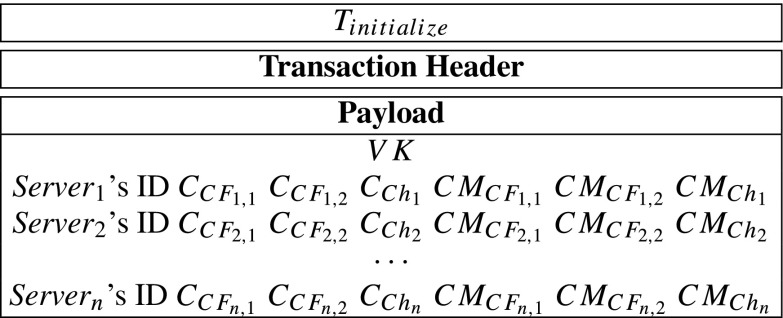
 
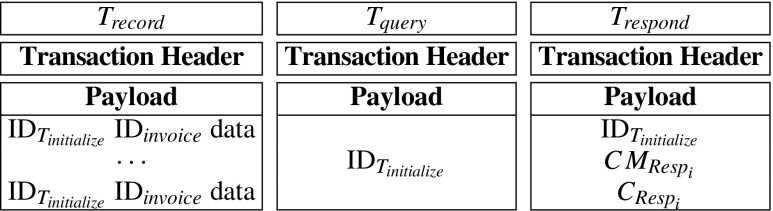


In MIStore, time is also divided into epoches. In each epoch, record-nodes will generate a block belong to the epoch via selected consensus scheme, and a block can be described as follow (Table 2):

**Table 2:** Format of block

Block header	
Name	Description
Version	Block version number
Hash	The block’s hash value
Parent hash	The previous block’s hash value
Difficulty	The proof-of-work target difficulty
Timestamp	Creation time of the block
Merkle root	The root of Merkle Tree of transactions
Nonce	A random counter for proof-of-work
Block body: Transactions	
Transaction_1_, Transaction_2_⋯, Transaction_*n*_	

Besides, record-nodes are responsible to verify all publicly verifiable data of transactions before the transactions are included in the blockchain. If any publicly verifiable data is invalid, then honest record-nodes will reject corresponding transactions. The transaction then will not be included in the blockchain. Moreover, due to adopting the Practical Byzantine Fault-tolerance consensus scheme, if a transaction has presented at the blockchain, then all nodes can consider that the transaction’s publicly verifiable data is credible. Therefore, other nodes can trust the transaction’s publicly verifiable data without any other verifications.

Futhermore, in the MIStore system, record-nodes may perform two kinds of verifications on transactions. The first one is the *basic verification*, which should be performed on all transactions. They are:

- The transaction’s inputs have not been used previously.
- The transaction’s signature is valid.
- The sum of input coins is equal to the sum of output coins.

**Fig. 2:**
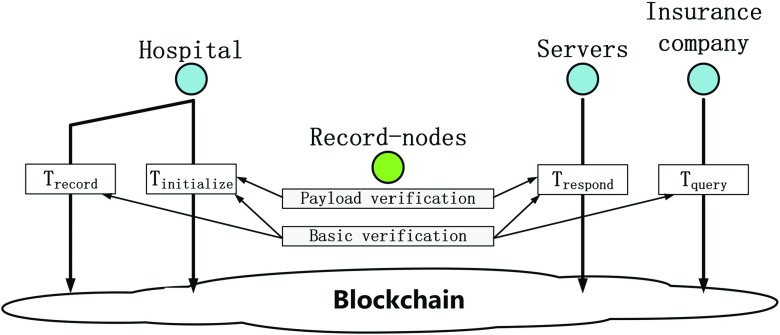
Basic verifications and payload verifications. All transactions are verified by record-nodes before they are recorded in the blockchain. For query-transactions and record-transactions, record-nodes just perform basic verifications. While for initialize-transactions and respond-transactions, record-nodes perform basic verifications and payload verifications

The second one is the *payload verification*, which can be performed on initialize-transactions and respond-transactions. It means that, in the payloads of initialize-transaction and respond-transaction, there is publicly verifiable data that may be verified by record-nodes. If a transaction has presented at the blockchain, then it means that most of record-nodes have accepted the transaction’s publicly verifiable data. Therefore, the transaction’s receiver can consider that the transaction’s publicly verifiable data is credible. Thus the receiver just needs to perform some other verifications that can be performed only by him. In this way, the most of verification computations are performed by record-nodes and it helps to decreases servers’ and insurance company’s verification computations significantly. Figure 2 describes verifications of initialize-transaction, record-transaction, query-transaction and respond-transaction.

### Construction of MIStore

To clearly introduce the MIStore system, in this subsection, we describe the basic instance that contains a hospital, a patient, an insurance company and *n* servers. Specifically, *Sr*_1_, *Sr*_2_, ⋯ , *Sr*_*n*_ denote *n* servers’ IDs, ID_*H*_ is the hospital’s ID, ID_*P*_ is the patient’s ID and ID_*I*_ describes the insurance company’s ID. Essentially, more complex instance can be constructed with the basic instance. The symbols used in the paper are shown in Table 3.

**Table 3:** Symbols of MIStore

Symbol	Description
*g*	The generator of a cyclic group $\mathbb {}$
*e*	The bilinear map, *e*: $\mathbb {G} \times \mathbb {G} \rightarrow \mathbb {G}$
$\mathbb {F}_{p}$	The finite field with character *p*
*I* *D*_*H*_	The hospital’s ID
*S* *r*_*i*_	The *i*-th servers’ IDs
*I* *D*_*I*_	The insurance company’s ID
$ID_{T_{i}}$	The transaction *T*_*i*_’s ID
*I* *D*_*P*_	The patient’s ID
*VK*	The verification key
{*pk*_*H*_, *sk*_*H*_}	The hospital’s key pair
{*pk*_*i*_, *sk*_*i*_}	The *i*-th server’s key pair, for *i* from 1 to *n*
{*pk*_*I*_, *sk*_*I*_}	The insurance company’s key pair
*d*_*i*_	The *i*-th plaintext message protected by *n* servers
{*CF*_*i*,1_, *CF*_*i*,2_, *Ch*_*i*_}	The *i*-th server’s core-share
*R* *e* *s* *p*_*i*_	The *i*-th server’s respnse

At first, the hospital and *n* servers should have published smart contracts to mortgage a certain amount of guarantee coins at the blockchain, respectively. If someone publishes some incorrect data that is verifiable, then the discoverer can send the corresponding evidences to the bumbler’s smart contract to prove that the bumbler sent an incorrect data. Then, the discoverer can automatically obtain a amount of reward from the bumbler’s smart contract.

After mortgaging guarantee coins, the MIStore system can be performed as follows:

- **Step 1: Initialization.** Hospital randomly samples two polynomials *F*_1_(*x*) and *F*_2_(*x*) of degree *t* − 1 over $\mathbb {F}_{p}$ as the following polynomials:
  $$F_{1}(x)=a_{t-1}x^{t-1}+a_{t-2}x^{t-2}+{\cdots} +a_{1}x+s_{core,1}, $$
  $$F_{2}(x)=d_{t-1}x^{t-1}+d_{t-2}x^{t-2}+{\cdots} +d_{1}x+s_{core,2}, $$
  where $s_{core,1},s_{core,2},a_{1},\cdots ,a_{t-1},d_{1},\cdots ,d_{t-1}\in \mathbb {F}_{p}$, *a*_*t*− 1_≠ 0 and *d*_*t*− 1_≠ 0. Let
  $$f_{1}(x)=a_{t-1}x^{t-1}+a_{t-2}x^{t-2}+\cdots+a_{1}x, $$
  $$f_{2}(x)=d_{t-1}x^{t-1}+d_{t-2}x^{t-2}+\cdots+d_{1}x. $$
  Then, we have *F*_1_(*x*) = *f*_1_(*x*) + *s*_*core*,1_ and *F*_2_(*x*) = *f*_2_(*x*) + *s*_*core*,2_. Hospital computes
  $$f_{1}(x)f_{2}(x)=q_{2t-2}x^{2t-2}+q_{2t-3}x^{2t-3}+\cdots+q_{2}x^{2}. $$
  After that, hospital randomly samples *l*(*x*) of degree *t* − 1 from $\mathbb {F}_{p}[x]$ as follow:
  $$l(x)=c_{t-1}x^{t-1}+c_{t-2}x^{t-2}+{\cdots} +c_{1}x. $$
  Let
  $$h(x)=f_{1}(x)f_{2}(x)-l(x)=b_{2t-2}x^{2t-2}+b_{2t-3}x^{2t-3}+\cdots+b_{1}x. $$
  Then hospital generates a verification key *VK* as follow:
  $$\begin{array}{@{}rcl@{}} \mathit{VK} &=& \{g,g^{a_{t-1}},\cdots,g^{a_{1}},g^{s_{core,1}},g^{d_{t-1}},\cdots,g^{d_{1}},g^{s_{core,2}},\\ && g^{b_{2t-2}}, \cdots,g^{b_{1}},g^{c_{t-1}},\cdots,g^{c_{1}}\}, \end{array} $$
  where *g* is a base point of 256-bit Barreto-Naehrig curve (BN-curve) [25]. For *i* from 1 to *n*, hospital does as follows:
  - Compute *CF*_*i*,1_ = *F*_1_(*Sr*_*i*_), *CF*_*i*,2_ = *F*_2_(*Sr*_*i*_) and *Ch*_*i*_ = *h*(*Sr*_*i*_). {*CF*_*i*,1_, *CF*_*i*,2_, *Ch*_*i*_} is *Server*_*i*_’s *core-share*.
  - Encrypts *CF*_*i*,1_, *CF*_*i*,2_, *Ch*_*i*_ with *Server*_*i*_’s public key *pk*_*i*_ into $C_{CF_{i,1}}=Enc_{pk_{i}}(CF_{i,1})$, $C_{CF_{i,2}}=Enc_{pk_{i}}(CF_{i,2})$ and $C_{Ch_{i}}=Enc_{pk_{i}}(Ch_{i})$ via ECIES. Only *Server*_*i*_ can decrypt them since only *Server*_*i*_ has the corresponding secret key *sk*_*i*_.
  - Compute commitments $CM_{CF_{i,1}}=g^{CF_{i,1}}$, $CM_{CF_{i,2}}=g^{CF_{i,2}}$ and $CM_{Ch_{i}}=g^{Ch_{i}}$. The commitments will be used in later verifications without obtaining *CF*_*i*,1_, *CF*_*i*,2_ and *Ch*_*i*_.
  Then, the hospital generates a initialize-transaction *T*_*initialize*_ as follows: 
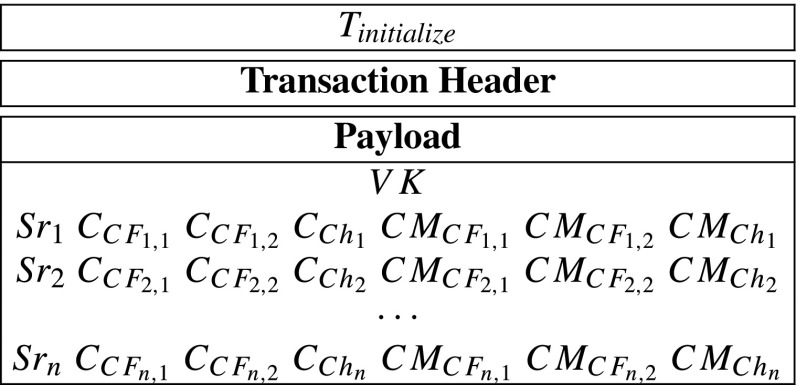

  After that, the hospital sends the *T*_*initialize*_ to blockchain network.
- **Step 2: Record-nodes verify** *T*_*initialize*_. Honest record-nodes will verify all new initialize-transactions before appending them at the blockchain. For instance, when an honest record-node receives the *T*_*initialize*_, he will verify its verification key (*VK*) at first, and then verify other data with the *VK*. If *T*_*initialize*_ passes the verifications, then the record-node accepts the *T*_*initialize*_ and writes it in his local block, otherwise, he will reject the *T*_*initialize*_. The verifications are described as follows:
  - First, verify the verification key *VK*. The record-node verifies whether polynomials *f*_1_(*x*), *f*_2_(*x*), *h*(*x*) and *l*(*x*), committed in verification key, are well-formed. Specifically, the record-node does as follows:
    - Randomly sample a number $x_{0}\in \mathbb {F}_{p}$.
    - Compute
      $$\begin{array}{@{}rcl@{}} g_{1}&=&(g^{a_{t-1}})^{x_{0}^{t-1}}(g^{a_{t-2}})^{x_{0}^{t-2}}{\cdots} (g^{a_{1}})^{x_{0}}\\ &=& g^{a_{t-1}x_{0}^{t-1}+a_{t-2}x_{0}^{t-2}+{\cdots} +a_{1}{x_{0}}}\\ g_{2}&=&(g^{d_{t-1}})^{x_{0}^{t-1}}(g^{d_{t-2}})^{x_{0}^{t-2}}{\cdots} (g^{d_{1}})^{x_{0}}\\ &=& g^{d_{t-1}x_{0}^{t-1}+d_{t-2}x_{0}^{t-2}+{\cdots} +d_{1}{x_{0}}}\\ g_{3}&=&(g^{b_{2t-2}})^{x_{0}^{2t-2}}(g^{b_{2t-3}})^{x_{0}^{2t-3}}{\cdots} (g^{b_{1}})^{x_{0}}\\ &=& g^{b_{2t-2}x_{0}^{2t-2}+b_{2t-3}x_{0}^{2t-3}+{\cdots} +b_{1}x_{0}}\\ g_{4}&=&(g^{c_{t-1}})^{x_{0}^{t-1}}(g^{c_{t-2}})^{x_{0}^{t-2}}{\cdots} (g^{c_{1}})^{x_{0}}\\ &=& g^{c_{t-1}x_{0}^{t-1}+c_{t-2}x_{0}^{t-2}+{\cdots} +c_{1}x_{0}} \end{array} $$
    - If
      $$e(g_{1},g_{2})=e(g_{3}g_{4},g), $$
      then the record-node accepts that *f*_1_(*x*), *f*_2_(*x*), *h*(*x*) and *l*(*x*) satisfy relationships and forms mentioned at **Step 1**. Otherwise he rejects the *T*_*initialize*_ and stops his verifications.
  - Second, verify commitments $CM_{CF_{i,1}}$, $CM_{CF_{i,2}}$, $CM_{Ch_{i}}$, *i* from 1 to *n*. Specifically, the record-node computes as follows:
    - Compute
      $$\begin{array}{@{}rcl@{}} {CF}_{i,1}^{*} &=& (g^{a_{t-1}})^{Sr_{i}^{t-1}}{\cdots} (g^{a_{1}})^{Sr_{i}}(g^{s_{core,1}})\\ {CF}_{i,2}^{*} &=& (g^{d_{t-1}})^{Sr_{i}^{t-1}}{\cdots} (g^{d_{1}})^{Sr_{i}}(g^{s_{core,2}})\\ {Ch}_{i}^{*} &=& (g^{b_{2t-2}})^{Sr_{i}^{2t-2}}{\cdots} (g^{b_{1}})^{Sr_{i}} \end{array} $$
    - If
      1 $$ {CF}_{i,1}^{*} = CM_{CF_{i,1}}, {CF}_{i,2}^{*} = CM_{CF_{i,2}} \text{ and } {Ch}_{i}^{*} = CM_{Ch_{i}}, $$
      then the record-node accepts that $CM_{CF_{i,1}}$, $CM_{CF_{i,2}}$ and $CM_{Ch_{i}}$ are correctly computed by the hospital, otherwise he rejects the *T*_*initialize*_ and stop his verifications.
  If any data cannot pass corresponding verification, then the record-node rejects the *T*_*initialize*_.

#### Remark 1

Because the record-node randomly samples the number *x*_0_, so the Eq. 1 is enough to prove the validation of the verification key.

- **Step 3: Servers verify core-shares**. *i* from 1 to *n*, when the *Server*_*i*_ sees the *T*_*initialize*_ at the blockchain, the server may perform the following computations:
  - Decrypt $C_{CF_{i,1}}$, $C_{CF_{i,2}}$ and $C_{Ch_{i}}$. Then he obtains *CF*_*i*,1_, *CF*_*i*,2_ and *Ch*_*i*_.
  - If
    $$CM_{CF_{i,1}}=g^{CF_{i,1}},CM_{CF_{i,2}}=g^{CF_{i,2}}\textrm{ and }CM_{Ch_{i}}=g^{Ch_{i}}, $$
    then the server accepts that the *T*_*initialize*_ is valid, otherwise he can send his evidences $\text {ID}_{T_{initialize}}$, *CF*_*i*_ and *Ch*_*i*_ to the hospital’s smart contract. After that, the server can obtain a amount of reward.
- **Step 4: Record.** After seeing the *T*_*initialize*_ at the blockchain, the hospital may generate record-transactions. Moreover, let *da*_1_, *da*_2_, ⋯, *da*_*m*_ denote the patient’s spending records. The each spending record has a unique invoice number, ${\text {ID}}_{invoice}^{i}$. However, they belong to the same initialize-transaction *T*_*initialize*_. Without loss of generality, we assume that the hospital generates two record-transactions (*T*_1_ and *T*_2_), and the patient’s spending records are *da*_1_, *da*_2_, *da*_3_, *da*_4_. Then, *i* from 1 to 4, hospital randomly divides *da*_*i*_ into *da*_*i*_ = *dd*_*i*,1_*dd*_*i*,2_. Then, the hospital computes
  $$\mathsf{s}_{i,1} = \mathsf{dd}_{i,1} - \mathsf{s}_{core,1}, \mathsf{s}_{i,2}=\mathsf{dd}_{i,2}-\mathsf{s}_{core,2}. $$
  Then, it generates transactions *T*_1_, *T*_2_ as follows: 
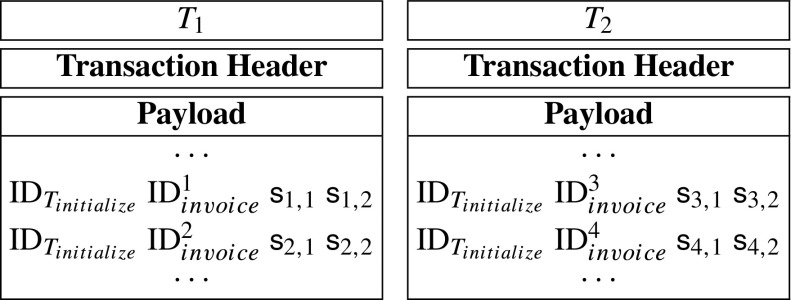

  After that, the hospital sends *T*_1_, *T*_2_ to blockchain network.
- **Step 4.5: Patient verifies spending records.** The patient knows the true spending records *da*_1_, *da*_2_, *da*_3_, *da*_4_. After seeing the transactions *T*_1_ and *T*_2_ at the blockchain, he can verify the correctness of his spending data of *T*_1_ and *T*_2_. Specifically, he verify whether the following equations are correct, *i* from 1 to 4.
  $$e(g^{\mathsf{da}_{i}},g)=e(g^{s_{core,1}}g^{\mathsf{s}_{i,1}},g^{s_{core,2}}g^{\mathsf{s}_{i,2}}) $$
  If the above equation holds for each *i* from 1 to 4, then the patient considers that his spending data is correctly processed by the hospital. Otherwise, he will consider that the hospital is dishonest and send the evidences to the hospital’s smart contract to get a certain number of reward.
- **Step 5: Query.** When the insurance company wants to get a sum of spending records related to the initialize-transaction *T*_*initialize*_, he may send a query-transaction *T*_*query*_ containing ID$_{T_{\mathit {initialize}}}$ to the blockchain network. The *T*_*query*_ is described as follows: 
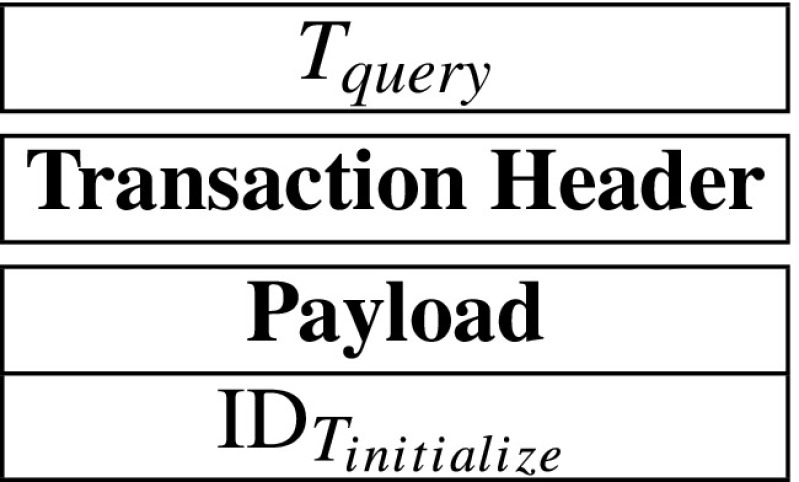

  When the query-transaction is appended at the blockchain, it means that insurance company wants to know the sum of all spending records of the patient corresponding to ID$_{T_{initialize}}$ until now.

#### Remark 2

After the *T*_*query*_ is correctly responded by at least t servers, when the patient performs new payments with the hospital, the hospital should generates another new initialize-transaction, including a new verification key, for the patient.

- **Step 6: Respond.** After the *T*_*query*_ has presented at blockchain. If a server wishes to respond the query, then he will generate a response according to the *T*_*query*_. After that, the server will secretly send his response to the insurance company via a respond-transaction *T*_*respond*_. If insurance company collets at least *t* responses correctly computed by corresponding servers, then insurance company can recover the correct sum of spending records related to the ID_*initialize*_. To introduce the process, without loss of generality, we assume that the *t* servers are *Server*_1_, *Server*_2_, ⋯ , *Server*_*t*_ and they wish to respond the *T*_*query*_. According to *T*_*query*_, the servers can obtain *s*_1,1_, *s*_1,2_, *s*_2,1_, *s*_2,2_, *s*_3,1_, *s*_3,2_, *s*_4,1_, *s*_4,2_ which are recorded in *T*_1_ and *T*_2_. First, *i* from 1 to *t*, the *Server*_*i*_ computes as follows:
  $$\mathit{Resp}_{i} = \sum\limits_{j = 1}^{4} (CF_{i,1} + s_{j,1})(CF_{i,2}+s_{j,2})-4\cdot Ch_{i}. $$
  Then *Server*_*i*_ encrypts *Resp*_*i*_ into
  $$C_{\mathit{Resp}_{i}} = Enc_{pk_{I}}(\mathit{Resp}_{i}) $$
  with insurance company’s public key *pk*_*I*_. Then *Server*_*i*_ computes a commitment of *Resp*_*i*_ as follow:
  $$CM_{\mathit{Resp}_{i}} = g^{\mathit{Resp}_{i}}. $$
  After that, *Server*_*i*_ generates a respond-transaction ${T}_{\mathit {respond}}^{i}$, containing $\text {ID}_{T_{initialize}}$, $CM_{\mathit {Resp}_{i}}$ and $C_{\mathit {Resp}_{i}}$. The *T*_*respond*_ can be described as follow: 
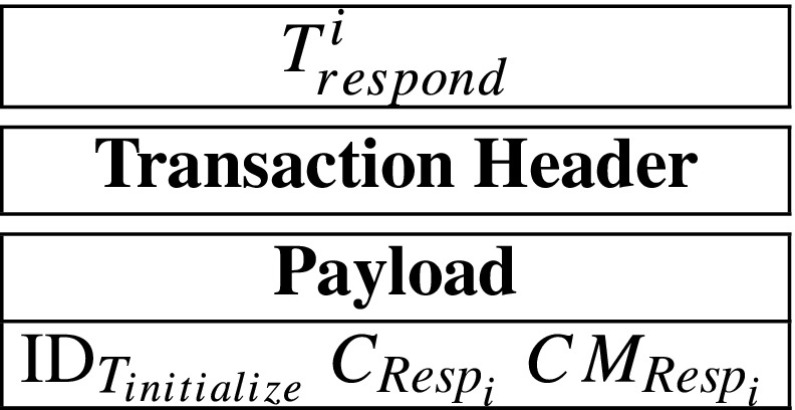

  Overall, servers *Server*_1_, *Server*_2_, ⋯, *Server*_*t*_ will generate $C_{\mathit {Resp}_{1}}$, $C_{\mathit {Resp}_{2}}$, ⋯, $C_{\mathit {Resp}_{t}}$ and $CM_{\mathit {Resp}_{1}}$, $CM_{\mathit {Resp}_{2}}$, ⋯, $CM_{\mathit {Resp}_{t}}$. Then, *Server*_1_, *Server*_2_, ⋯, *Server*_*t*_ generate transactions ${T}_{\mathit {respond}}^{1}$, ${T}_{\mathit {respond}}^{2}$, ⋯, ${T}_{\mathit {respond}}^{t}$, respectively. Because only the insurance company has the corresponding secret key *sk*_*I*_, so only the insurance company can decrypt $C_{\mathit {Resp}_{1}}$, $C_{\mathit {Resp}_{2}}$, ⋯, $C_{\mathit {Resp}_{t}}$. After that, the servers send ${T}_{\mathit {respond}}^{1}$, ${T}_{\mathit {respond}}^{2}$, ⋯, ${T}_{\mathit {respond}}^{t}$ to the blockchain network.
- **Step 7: Record-nodes verify** ${T}_{\mathit {respond}}^{1}$, ${T}_{\mathit {respond}}^{2}$, ⋯, ${T}_{\mathit {respond}}^{t}$. After receiving the respond-transactions ${T}_{\mathit {respond}}^{1}$, ${T}_{\mathit {respond}}^{2}$, ⋯, ${T}_{\mathit {respond}}^{t}$, a record-node may verify validations of their $CM_{\mathit {Resp}_{1}}$, $CM_{\mathit {Resp}_{2}}$ and $CM_{\mathit {Resp}_{t}}$. Specifically, *i* from 1 to *t*, the record-node performs as follows:
  - Compute
    $$g^{CF_{i}} = (g^{a_{t-1}})^{Sr_{i}^{t-1}} {\cdots} (g^{a_{1}})^{Sr_{i}} (g^{a_{0}}) = g^{a_{t-1}Sr_{i}^{t-1}+\cdots+a_{1}Sr_{i}+s_{core}} $$
    $$g^{Ch_{i}}=(g^{b_{2t-2}})^{Sr_{i}^{2t-2}}{\cdots} (g^{b_{1}})^{Sr_{i}}=g^{b_{2t-2}Sr_{i}^{2t-2}+\cdots+b_{1}Sr_{i}} $$
  - With *s*_1,1_, *s*_1,2_, *s*_2,1_, *s*_2,2_, *s*_3,1_, *s*_3,2_, *s*_4,1_, *s*_4,2_ and the bilinear map *e*, the record-node further computes
    $$\begin{array}{@{}rcl@{}} E_{i} &=& e(g^{CF_{i,1}}g^{\mathsf{s}_{1,1}},g^{CF_{i,2}}g^{\mathsf{s}_{1,2}})\\ && \times e(g^{CF_{i,1}}g^{\mathsf{s}_{2,1}},g^{CF_{i,2}}g^{\mathsf{s}_{2,2}})\\ && e(g^{CF_{i,1}}g^{\mathsf{s}_{3,1}},g^{CF_{i,2}}g^{\mathsf{s}_{3,2}})\\ && \times e(g^{CF_{i,1}}g^{\mathsf{s}_{4,1}},g^{CF_{i,2}}g^{\mathsf{s}_{4,2}}) \end{array} $$
  - If
    $$E_{i}/e(g^{Ch_{i}},g^{4})=e(CM_{\mathit{Resp}_{i}},g), $$
    then the record-node considers that $CM_{\mathit {Resp}_{i}}$ is valid.
- **Step 8: Recover.** Because the ${T}_{\mathit {respond}}^{1}$, ${T}_{\mathit {respond}}^{2}$, ⋯, ${T}_{\mathit {respond}}^{t}$ present at the blockchain, it means that the transactions pass all previous all verifications. Therefore, the insurance company just needs to perform the final verification that can by performed only by him. That is, *i* from 1 to *t*, the insurance company decrypts $C_{\mathit {Resp}_{i}}$ and then obtain *Resp*_*i*_. If
  $$CM_{\mathit{Resp}_{i}}=g^{\mathit{Resp}_{i}}, $$
  then insurance company accepts that the *Resp*_*i*_ is correctly computed by *Server*_*i*_. Otherwise he rejects the response and can send his evidences ID$_{T_{\mathit {respond}}}$ and *Resp*_*i*_ to the *Server*_*i*_’s smart contract, and then insurance company can obtain a amount of reward. If all the *t* responses pass the verifications, then the insurance company uses *lagrange interpolation* to reconstruct a polynomial as follow:
  $$\tilde{F}(x) = \sum\limits_{i = 1}^{t} \mathit{Resp}_{i} \prod\limits_{j = 1,j\neq i}^{t} \frac{x-Sr_{j}}{Sr_{i}-Sr_{j}}. $$
  Finally, the insurance company calculates $\tilde {F}(0)$ that is the desired result.

## Performance evaluation

In this section, we evaluate a performance of the MIStore system. The performance evaluation can be broken into three parts. The first part studies the processing time of cryptographic and mathematic computations in this system. The time of processing transactions is researched in the second part. The last part further demonstrates the processing time of blocks when different transactions are sent to the blockchain network. The section starts with the prototype system setting.

### Prototype system setting

MIStore’s efficiency mainly depends on the blockchain platform and performance of cryptographic schemes. For instance, in the paper, we use the Ethererum blockchain as the blockchain platform. Specifically, Ethereum’s block can contains transactions of at most 62,360 bytes, its average block interval is about 15 s and its transaction’s payload contains at most 1014-byte data, so the MIStore’s efficiency is significantly limited by the Ethererum blockchain. Therefore, if we use some other more suitable blockchain, then we might get a better throughput. Besides, we use our BN-curve code to perform the pairing and point multiplication. Therefore, time cost of pairing and point multiplication may be longer than the previous optimal works. For instance, in Pinocchio [34], due to their excellent code, a pairing computation just takes 0.9 ms, while ours takes about 84.651 ms. Therefore, if we use their computer platform and code, maybe the performance of the prototype system could be improved.

We implement a prototype system that is a (2,3)-threshold MIStore protocol among three servers. Specifically, it contains a hospital, a patient, an insurance company and three servers. We use laptops and virtual machines to perform the prototype system. Our laptop’s configuration is described as follows: the Intel i5-5300 CPU with 2.30GHz, 4GB memory, Windows 10 OS. In the local area network, we deploy a local blockchain via go-ethereum that is a Go implementation of the Ethereum protocol (https://github.com/ethereum/go-ethereum). In the blockchain network, we deploy four record-nodes (miners), and we use transaction simulator (https://github.com/ethereum/go-ethereum) to simulate the hospital, servers and insurance company to generate and send transactions. Moreover, we record MIStore system’s data in the transaction’s payload. In the Ethereum blockchain, a transaction’s payload can record data of at most 1014 bytes.

Additionally, Ethererum has a embedded signature scheme that is the ECDSA with the secp256k1 elliptic curve [26]. For convenience, we use the scheme to sign messages. Besides, to encrypt key data recorded in the payloads of initialize-transaction and respond-transaction, we use the encryption scheme ECIES with the elliptic curve secp256k1 to encrypt the key data via receiver’s public key. It results in that each encrypted message has a length of 96 bytes. Moreover, the encrypted data can be decrypted only by the corresponding receivers since only he has the corresponding private key.

Furthermore, for committing data and verifying committed data, we utilize 256-bit Barreto-Naehrig curve (BN-curve) [25] to commit the data via the base point multiplication. For instance, let *G* be the base point of the BN-curve. Then, the secret *s* can be committed by *sG*. Therefore, a commitment has a length of 64 bytes since any point of the BN-curve has two coordinates and each of the coordinates is of 32 bytes. Moreover, we use the bilinear map *e* (pairing computation) constructed by the BN-curve to verify the correctness of the commitments. Specifically, *e*(*g*^*a*^, *g*^*b*^) = *e*(*g*^*ab*^, *g*). For instance, if we want to verify *ab* = *c* and we do not want to reveal *a*, *b* and c, then we may use the following equation to verify *ab* = *c*.

$$e(g^{a},g^{b})=e(g^{c},g). $$

### Processing time of cryptographic schemes

Generally, the time cost of performing cryptographic schemes will have a certain degree of influence on the time of processing transactions, and then it may influence the efficiency of the system. Therefore, in the sub-section, we discuss the processing time cost of cryptographic and mathematic components.

For each of encryption, decryption, point multiplication, point addition, signing, verifying signatures, pairing, field addition and field multiplication, we perform 1000 experiments to obtain their average time cost. Their average time cost is shown in Table 4.

**Table 4:** Average Time cost of cryptographic schemes

Scheme	Time cost
BN-curve Point Mul	29.569 ms
BN-curve Point Add	0.236 ms
Pairing	84.651 ms
Field Add	0.071 *μ* s
Field Mul	0.531 *μ* s
Secp256k1-curve ECDSA Sign	4.425 ms
Secp256k1-curve ECDSA Verify Sig	9.137 ms
Secp256k1-curve ECIES Encryption	8.745 ms
Secp256k1-curve ECIES Decryption	4.367 ms
Block Interval	15.2 s

### Generating transactions

In MIStore system, different transactions may have different payloads. For instance, an initialize-transaction includes a verification key, 9 commitments, 3 servers’ IDs and 9 encrypted messages, while a respond-transaction only contains a initialize-transaction’s ID, an encrypted response and a commitment about the response. Moreover, the sizes of payloads of initialize-transaction, query-transaction and respond-transaction are fixed, while the size of payload of record-transaction is variable. Therefore, different transactions may have different generation time. In the sub-section, we study generation time of transactions in the implementation. We discuss the initialize-transaction at first.

In our prototype system, according to “??”, a *T*_*initialize*_’s payload includes a verification key, three servers’ IDs, 9 encrypted messages and 9 commitments. Moreover, according to “??”, the data recorded in the *T*_*initialize*_’s payload is of 1504 bytes. However, the payload of a transaction, in the Ethererum blockchain, can include at most 1014 bytes. In other words, one transaction cannot contain 1504 bytes. Therefore, in the prototype system, we divide the *T*_*initialize*_ into ${T}_{\mathit {initialize}}^{1}$, ${T}_{\mathit {initialize}}^{2}$ and ${T}_{\mathit {initialize}}^{3}$ in order to record all its data. Specifically, the both transactions can be described as follows: 
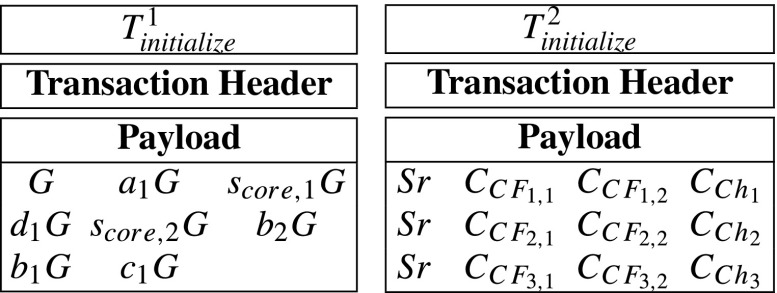
 
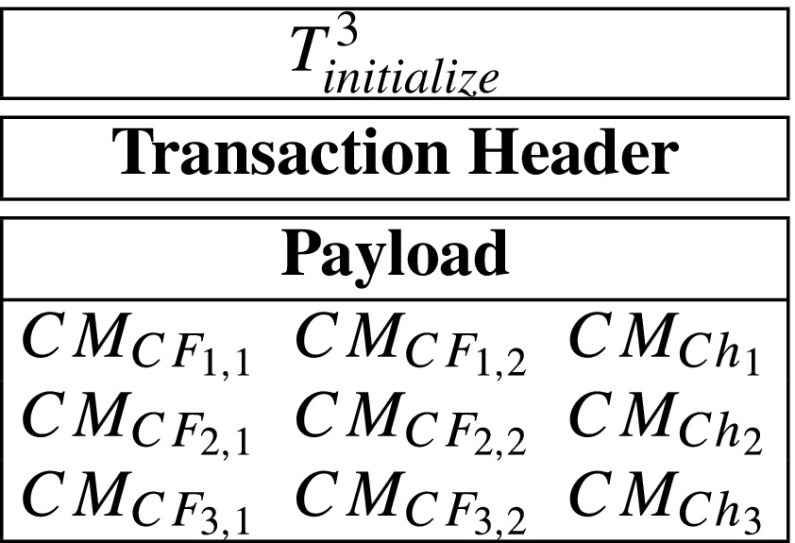


Indeed, record-transactions may have payloads with variable sizes. Moreover, a basic message recorded in a record-transaction’s payload is a array of {ID_*initialize*_, ID_*invoice*_, *s*_*k*,1_, *s*_*k*,2_} which is of 128 bytes. Due to that the a payload can include at most 1014 bytes, a record-transaction’s payload can contain at most 7 × 128 = 896 bytes. For convenience, in the prototype system, we only generate record-transactions with the largest payload. Specifically, we only generate two kinds of record-transactions which are described as follows: 
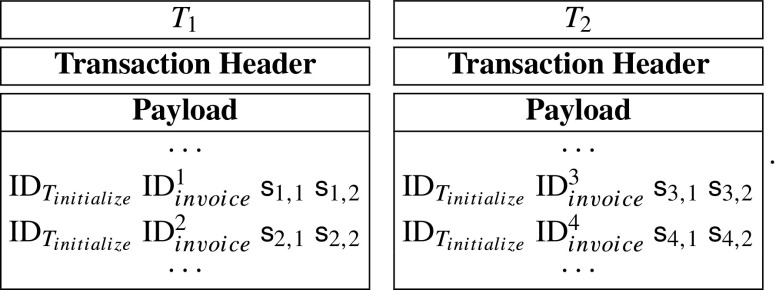


A query-transaction, whose payload just includes a initialize-transaction’s ID, can be shown as follows: 
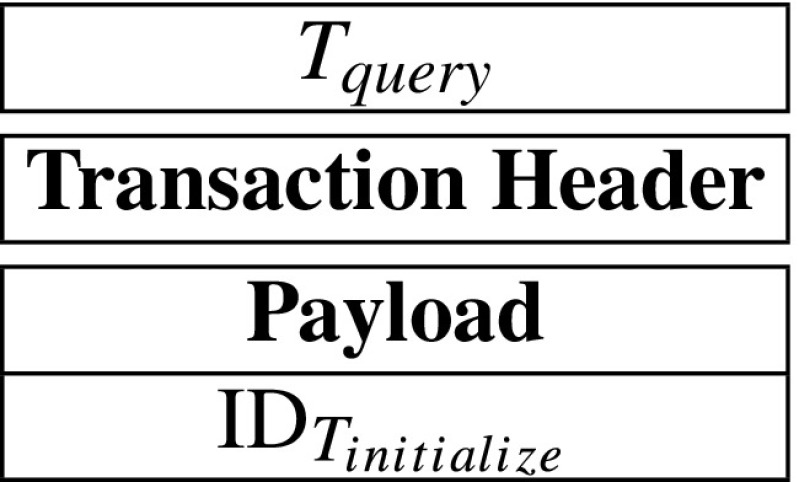


A respond-transaction’s payload contains the corresponding initialize-transaction’s ID, an encrypted response and a commitment of the response. Specifically, *Server*_*i*_’s respond-transaction can be described as follow: 
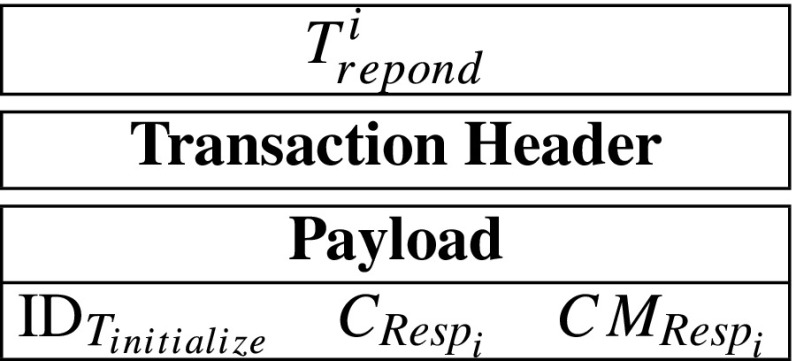


In the prototype system, sizes of transactions’ payloads are shown in Table 5. For each of ${T}_{\mathit {initialize}}^{1}$, ${T}_{\mathit {initialize}}^{2}$, ${T}_{\mathit {initialize}}^{3}$, *T*_*record*_, *T*_*query*_ and *T*_*respond*_, we generate 1000 transactions in order to obtain their average generating time cost. Then their average time cost are shown in Table 6.

**Table 5:** Payloads of transactions used in our prototype system

Payload	Content	Size
Payload of ${T}_{\mathit {initialize}}^{1}$	A verification key	512 bytes
Payload of ${T}_{\mathit {initialize}}^{2}$	3 IDs, 9 encrypted messages	960 bytes
Payload of ${T}_{\mathit {initialize}}^{3}$	9 commitments	576 bytes
Payload of *T*_*record*_	1 to 7 arrays of spending data	128-896 bytes
Payload of *T*_*query*_	1 ID	32 bytes
Payload of *T*_*respond*_	A hospital’s ID, a encrypted response, a commitment	192 bytes

**Table 6:** Average time cost of processing transactions

Operation on transaction	Computations	Time cost
Hospital generates a ${T}_{\mathit {initialize}}^{1}$	1S + 7PM	212.209 ms
Hospital generates a ${T}_{\mathit {initialize}}^{2}$	1S + 9E	83.931 ms
Hospital generates a ${T}_{\mathit {initialize}}^{3}$	1S + 9PM	271.347 ms
Hospital generates a *T*_*record*_	1S + 14FA	5.226 ms
Insurance company generates a *T*_*query*_	1S	5.226 ms
Server generates a *T*_*respond*_	1S + 5FM + 12FA + 1E + 1PM	43.543 ms
Record-node verifies a ${T}_{\mathit {initialize}}^{1}$	1V + 5PM + 2PA	157.454 ms
Record-node verifies a ${T}_{\mathit {initialize}}^{2}$	1V	9.137 ms
Record-node verifies a ${T}_{\mathit {initialize}}^{3}$	1V + 15PM + 9PA	454.796 ms
Record-node verifies a *T*_*record*_	1V	9.137 ms
Record-node verifies a *T*_*query*_	1V	9.137 ms
Record-node verifies a *T*_*respond*_	1V + 2PM + 10PA + 5Pairing	494.361 ms
Server verifies a ${T}_{\mathit {initialize}}^{2}$	3D+ + 3PM	101.807 ms
Insurance company verifies a *T*_*respond*_	1D + 1PM	33.936 ms
Insurance company recovers the result	4FA + 4FM	< 0.005 ms

In the table, *S* is a signing computation, *V* denotes a signature verification, *PM* describes a point multiplication on the ECC, *PA* is a point addition on the ECC, *Pairing* means a pairing computation, *E* is a encryption, *D* denotes a decryption, *FM* describes a field multiplication and *FA* is a field addition. For instance, “2PM + 3PA + 1V + 6Pairing” denotes that the corresponding computations contain 2 point multiplications, 3 point additions, 1 signature verification and 6 pairing computations

### Verifying transactions

In the system, before a transaction is appended at the blockchain, most record-nodes must verify the transaction. Specifically, record-nodes verify all publicly verifiable data of the transaction. Moreover, if a transaction has appeared at the blockchain, then it means that it has been accepted by most record-nodes. Therefore, the transaction’s publicly verifiable data is credible. Consequently, others (e.g., hospital, patient, insurance company and servers ) do not have to verify the transaction’s publicly verifiable data. In this way, it significantly reduces verifying computations of users. In the sub-section, we study transactions’ verification time cost. All publicly verifiable data of transactions are summarized as follows:

- All transactions’ signatures are publicly verifiable data that can be verified by record-nodes. Therefore, if a transaction has appeared at the blockchain, then the transaction’s signature is credible, and others need not to verify the signature.
- Except signatures, the payloads of initialize-transaction and respond-transaction have public verifiable data that can be verified by record-nodes. Specifically, they are the initialize-transaction’s verification key, commitments of core-shares and commitments of responses. Consequently, if an initialize-transaction (or a query-transaction) has appeared at the blockchain, then its publicly verifiable data is credible. Therefore, the transaction’s receiver need not to verify the public verifiable data.

In this way, the transaction’s receiver just needs to verify some key data that can be verified by only him.

For each of ${T}_{\mathit {initialize}}^{1}$, ${T}_{\mathit {initialize}}^{2}$, ${T}_{\mathit {initialize}}^{3}$, *T*_*record*_, *T*_*query*_ and *T*_*respond*_, we verify 1000 transactions, and then obtain their average verifying time cost. Then their average verifying time cost are shown in Table 6. Specifically, in Table 6, *S* is a signing computation, *V* denotes a signature verification, *PM* describes a point multiplication on the ECC, *PA* is a point addition on the ECC, *Pairing* means a pairing computation, *E* is a encryption, *D* denotes a decryption, *FM* describes a field multiplication and *FA* is a field addition. For instance, “2PM + 3PA + 1V + 6Pairing” denotes that the corresponding computations contain 2 point multiplications, 3 point additions, 1 signature verification and 6 pairing computations.

It must be pointed that if the system does not use blockchain to record transactions and does not use record-nodes to help users to verify publicly verifiable data, then transaction receivers should perform more verifying computations than the blockchain-based system. Specifically, if that happens and we also use the above transactions and cryptographic schemes, then this will result in:

- Because all transactions are stored by some centralized nodes, so storages might be modified or deleted by the centralized nodes.
- All related users must independently verify all public verifiable data including the verification key and commitments.
- Servers and insurance company might be heavier than the blockchain-based system. Therefore, some computations and operations cannot be processed efficiently, even cannot be performed.

For instance, we assume that a non-blockchain-based MIStore (pure system) is performed. If an insurance company receives a respond-transaction, then it must verify all verifiable data, otherwise it will not trust the transaction. Specifically, he will costs about 528.297 ms to verify it. However, if the system is based on a blockchain network, that is the key point of the paper, then the insurance company just needs to cost 33.936 ms to verify some key data since other data has been verified by record-nodes. Comparisons between the pure system and the blockchain-based system are shown in Table 7. According to the Table 7, if the system is not based on the blockchain, then the insurance company and servers all need a certain amount of verifying computations. However, if the system is based on the blockchain, then most computations can be done by record-nodes, then the insurance company and servers just need to perform very few verifying computations.

**Table 7:** Comparisons between pure MIStore and blockchain-based MIStore

Comparative item	Time cost of pure MIStore			Time cost of blockchain-based MIStore			
	Hospital	Server	IC	Hospital	Server	IC	Record-node
Verifying ${T}_{\mathit {initialize}}^{1}$	0 ms	157.4 ms	157.4 ms	0 ms	0 ms	0 ms	157.4 ms
Verifying ${T}_{\mathit {initialize}}^{2}$	0 ms	110.9 ms	9.1 ms	0 ms	101.8 ms	0 ms	9.1 ms
Verifying ${T}_{\mathit {initialize}}^{3}$	0 ms	454.7 ms	454.7 ms	0 ms	0 ms	0 ms	454.7 ms
Verifying *T*_*record*_	0 ms	9.1 ms	9.1 ms	0 ms	0 ms	0 ms	9.1 ms
Verifying *T*_*query*_	0 ms	0 ms	9.1 ms	0 ms	0 ms	0 ms	9.1 ms
Verifying *T*_*respond*_	0 ms	0 ms	528.2 ms	0 ms	0 ms	33.9 ms	494.3 ms

IC denotes the insurance company

### Blockchain performance evaluation

We run our MIStore on the Ethereum blockchain. After generating a certain number of blocks, the block interval tends to be stable. That is, generating 1000 blocks takes about 4.3 h. In other words, generating a block takes about 15.2 s on average. Furthermore, in the Ethereum blockchain, a block can record transactions of at most 62,360 bytes, a transaction with an empty payload is of 308 bytes and a transaction’s payload can record data of at most 1014 bytes. Therefore, a transaction’s size should be from 308 bytes to 308 + 1014 = 1322 bytes.

According to Table 5 and above contents, in our implementation, any transaction’s size can be calculated. Transactions’ sizes are shown in Table 8. A block can record transactions of at most 62360 bytes. Therefore, if a block only record identical transactions, then the number of recorded transactions has a limit.

**Table 8:** Transactions’ sizes in our prototype system

Transaction	Size
${T}_{\mathit {initialize}}^{1}$	820 bytes
${T}_{\mathit {initialize}}^{2}$	1268 bytes
${T}_{\mathit {initialize}}^{3}$	884 bytes
*T*_*record*_	436-1204 bytes
*T*_*query*_	340 bytes
*T*_*respond*_	500 bytes

In our experiments, because different transactions have different significance, so the more significant transaction should be processed earlier. In the Ethererum blockchain, record-nodes (miners) earlier process a transaction with more transaction fee. Therefore, we set different transactions with different transaction fees. When transactions are pending in a record-node’s transaction pool, transactions with more fees will be recorded earlier. In the MIStore system, the initialize-transaction is the base of later transactions. Therefore, it should has the first priority. For quickly responding insurance company’s query, we set that query-transaction has the second priority and respond-transaction has the third priority. Finally, record-transaction has the lowest priority. In this way, the system’s responding rate will be obviously increased. In our experiments, their transaction fees are shown in Table 9.

**Table 9:** Transaction fee

Transaction	Transaction fee
${T}_{\mathit {initialize}}^{1}$	0.0001 ETH
${T}_{\mathit {initialize}}^{2}$	0.0001 ETH
${T}_{\mathit {initialize}}^{3}$	0.0001 ETH
*T*_*record*_	0.00001 ETH
*T*_*query*_	0.00006 ETH
*T*_*respond*_	0.00003 ETH

ETH denotes the unit of Ethererum coin

In our experiments, after an initialize-transaction has appeared at the blockchain, the hospital continually send record-transactions to the blockchain network. The record-transactions are same as mentioned at “??”. The data of record-transactions’ payloads is called as “spending-data”. Every block can contain at most 51 record-transactions with the most arrays of spending data. Then, a block can store spending-data of at most 45696 bytes. Because the blockchain generates a block per about 15 s on average, so the system can record spending-data of at most 3046.4 bytes per second on average. At some later time, the insurance company sends a query-transaction to the blockchain network. Consequently, in the next block, the insurance company can get 3 response-transactions. The respond-transactions are recorded by record-nodes earlier than record-transactions since it has larger transaction fee. Finally, the insurance company can recover his desired data. The whole process only takes about 24 s.

MIStore’s efficiency mainly depends on the blockchain platform. For instance, in the paper, we use the Ethererum blockchain as the platform. Specifically, Ethereum’s block can contains transactions of at most 62,360 bytes, its average block interval is about 15 s and its transaction’s payload contains at most 1014-byte data, so the MIStore’s efficiency is significantly limited by the blockchain platform. Therefore, if we use some other more suitable blockchain platform, then it might get a better throughput.

## Conclusion

In this paper, we propose a blockchain-based threshold medical insurance storage system, called MIStore. Because of combining with blockchain, the system obtains some special advantages, e.g., decentralization, tamper-resistance and record-nodes help users to verify publicly verifiable data. Firstly, the blockchain’s property of tamper-resistance gives users high-credibility. Moreover, due to the decentralization, users can communicate with each other without the third-parties. Secondly, the system supports the property of threshold. That is, patient’s data is confidentially controlled by servers specified by the hospital, and the stored data is always confidential for the servers as long as a certain number of the servers are honest. Furthermore, according to the insurance company’s query, the specified servers can perform homomorphic computations on the data and then get responses. If the insurance company can collect a threshold number of correct responses, then he can recover the correct patient’s spending data. Thirdly, all important data is verifiable. In particular, most of data is publicly verifiable. Therefore, record-nodes of blockchain can help users to perform the public verifications. Consequently, this significantly reduces users computations. Finally, a performance evaluation about the system is given.

## Figures and Tables

**Fig. 3:**
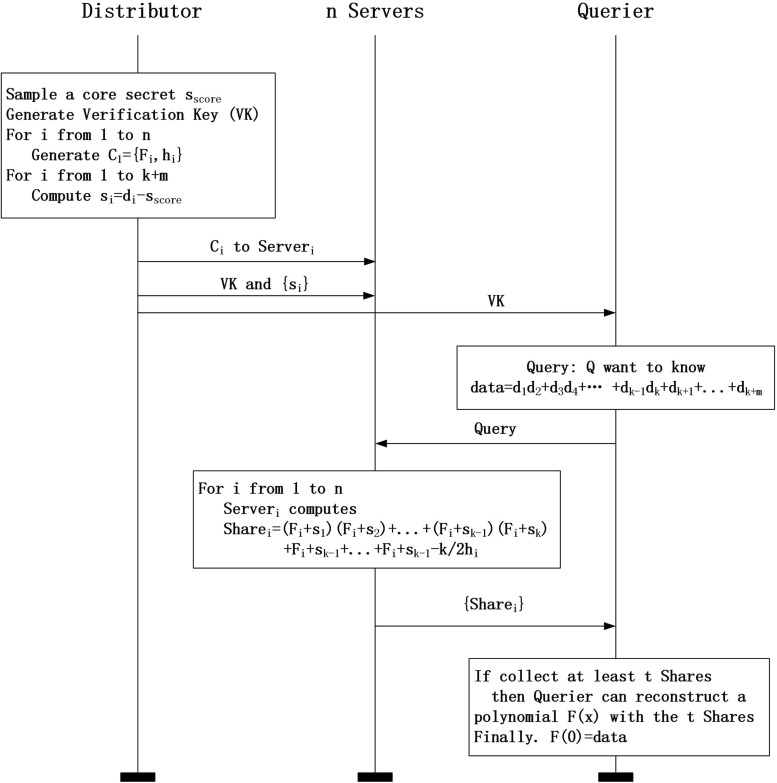
Work process of TVHCSS

## Appendix: (*t*, *n*)-threshold verifiable homomorphic confidential storage scheme

(*t*, *n*)-threshold verifiable homomorphic confidential storage scheme (TVHCSS) contains three parties (a distributor, *n* servers and a querier). Specifically, in TVHCSS, the distributor’s messages are protected and controlled by the *n* servers, and in each server’s hands, messages are ciphertext. When a querier sends a query to the *n* servers, if at least *t* servers return correct answers to the querier, then the querier can obtain what he wants. While if less than *t* servers return answers to the querier, then the querier cannot obtain anything.

### A.1 Construction of TVHCSS

Symbols, used in the scheme, are summarized at Table 10.

- *g* is a generator of a cyclic group 𝔾.
- *e* is a bilinear map, *e*: 𝔾 × 𝔾 → 𝔾. For instance, *e*(*g*^*a*^, *g*^*b*^) = *e*(*g*, *g*)^*a**b*^.
- *D* is the distributor’s ID.
- *S**r*_1_, *S**r*_2_, ⋯ , *S**r*_*n*_ denote *n* servers’ IDs.
- *Q* describes the querier’s ID.
- {*p**k*_*i*_, *s**k*_*i*_} denotes the *i*-th server’s key pair, for *i* from 1 to *n*.
- {*p**k*_*Q*_, *s**k*_*Q*_} is *Q*’s key pair.
- *d*_1_,*d*_2_, ⋯ ,*d*_*m*_ describe the plaintext messages that will be protected by *n* servers in ciphertext.
- $Sahre^{asr}_{i}$ is the *i*-the server’s answer.

**Table 10:** Symbols of TVHCSS

Symbol	Description
*g*	The generator of a cyclic group $\mathbb {G}$
*e*	The bilinear map, *e*: $\mathbb {G}\times \mathbb {G}\rightarrow \mathbb {G}$
$\mathbb {F}_{p}$	The finite field with character *p*
*D*	The distributor’s ID
*S* *r*_*i*_	The *i*-th servers’ IDs
*Q*	The querier’s ID
*VK*	The verification key
{*p**k*_*D*_,*s**k*_*D*_}	The *D*’s key pair
{*p**k*_*i*_,*s**k*_*i*_}	The *i*-th server’s key pair, for *i* from 1 to *n*
{*p**k*_*Q*_*s**k*_*Q*_}	The insurance company *I*’s key pair
*d*_*i*_	The *i*-th plaintext message protected by *n* servers
$CM_{F_{i}}$	$CM_{F_{i}}=g^{F_{i}}$
$CM_{h_{i}}$	$CM_{h_{i}}=g^{h_{i}}$
*S* *a* *h* *r* *e* *i*	The *i*-th server’s answer-share

The TVHCSS can be described as follows:

- **Initialize.** Let $\mathbb {F}_{p}$ be a finite field with character *p*. *D* randomly samples a polynomial *F*(*x*) of degree *t* − 1 over $\mathbb {F}_{p}$ as the following polynomial.
  $$F(x)=a_{t-1}x^{t-1}+a_{t-2}x^{t-2}+{\cdots} +a_{1}x+s_{core}, $$
  where $s_{core},a_{1},\cdots ,a_{t-1}\in \mathbb {F}_{p}$ and *a*_*t*− 1_ ≠ 0. We denote that *s*_*c**o**r**e*_ is the *core secret* in the system. Let
  $$f(x)=a_{t-1}x^{t-1}+a_{t-2}x^{t-2}+\cdots+a_{1}x. $$
  Then we have *F*(*x*) = *f*(*x*) + *a*_0_. *D* computes
  $$f(x)^{2}=q_{2t-2}x^{2t-2}+q_{2t-3}x^{2t-3}+\cdots+q_{2}x^{2}. $$
  After that, *D* randomly samples *l*(*x*) of degree *t* − 1 from $\mathbb {F}_{p}[x]$ as follow:
  $$l(x)=c_{t-1}x^{t-1}+c_{t-2}x^{t-2}+{\cdots} +c_{1}x. $$
  Let
  $$h(x)=f(x)^{2}-l(x)=b_{2t-2}x^{2t-2}+b_{2t-3}x^{2t-3}+\cdots+b_{1}x. $$
  *D* samples a generator *g* that can generate a cyclic group $\mathbb {G}$. Then *D* publishes a verification key *VK* as follow:
  $$\begin{array}{@{}rcl@{}} VK &=& \{g,g^{a_{t-1}},\cdots,g^{a_{1}},g^{s_{core}},g^{b_{2t-2}},g^{b_{2t-3}},\\ && \cdots,g^{b_{1}},g^{c_{t-1}},g^{c_{t-2}},\cdots,g^{c_{1}}\} \end{array} $$
- **Verify committed polynomials.** Anyone can verify whether polynomials *f*(*x*), *h*(*x*), *l*(*x*), committed in verification key, are well-formed and sound. Specifically, he can do as follows:
  - Randomly sample *t* different numbers $x_{0},x_{1},\cdots ,x_{t-1}\in \mathbb {F}_{p}$.
  - *j* from 0 to *t* − 1, compute
    $$\begin{array}{@{}rcl@{}} {g_{j}^{f}} &=& (g^{a_{t-1}})^{x_{j}^{t-1}}(g^{a_{t-2}})^{x_{j}^{t-2}}{\cdots} (g^{a_{1}})^{x_{j}}\\ &=& g^{a_{t-1}x_{j}^{t-1}+a_{t-2}x_{j}^{t-2}+{\cdots} +a_{t-1}{x_{j}}} \end{array} $$
    $$\begin{array}{@{}rcl@{}} {g_{j}^{h}} &=& (g^{b_{2t-2}})^{x_{j}^{2t-2}}(g^{b_{2t-3}})^{x_{j}^{2t-3}}{\cdots} (g^{b_{1}})^{x_{j}}\\ &=& g^{b_{2t-2}x_{j}^{2t-2}+b_{2t-3}x_{j}^{2t-3}+{\cdots} +b_{1}x_{j}} \end{array} $$
    $$\begin{array}{@{}rcl@{}} {g_{j}^{l}} &=& (g^{c_{t-1}})^{x_{j}^{t-1}}(g^{c_{t-2}})^{x_{j}^{t-2}}{\cdots} (g^{c_{1}})^{x_{j}}\\ &=& g^{c_{t-1}x_{j}^{t-1}+c_{t-2}x_{j}^{t-2}+{\cdots} +c_{1}x_{j}} \end{array} $$
  - If $e({g_{j}^{f}},{g_{j}^{f}})=e({g_{j}^{h}}{g_{j}^{l}},g)$, for all *j* from 0 to *t* − 1, then the verifier accepts that the polynomials, committed by verification key, are well-formed and sound. Otherwise, he rejects and return to step *Inilialize*.
- **Distribute.** *D* computes
  $$F_{i}=F(Sr_{i})\ and\ h_{i}=h(Sr_{i}). $$
  *D* encrypts {*F*_*i*_, *h*_*i*_} with *S**r*_*i*_’s public key as $C_{i}=Enc_{pk_{i}}(CF_{i},Ch_{i})$. *D* sends *C*_*i*_ to *S**r*_*i*_, respectively. For *C*_*i*_, only *S**r*_*i*_ can decrypt it since only *S*_*i*_ has the corresponding secret key. After obtaining *F*_*i*_ and *h*_*i*_, *S**r*_*i*_ can verify the soundness of {*F*_*i*_, *h*_*i*_} with verification key
  $$\begin{array}{@{}rcl@{}} &&\{g,g^{a_{t-1}},\cdots,g^{a_{1}},g^{s_{core}},g^{b_{2t-2}},g^{b_{2t-3}},\cdots,\\&&g^{b_{1}},g^{c_{t-1}},g^{c_{t-2}},\cdots,g^{c_{1}}\}. \end{array} $$
  Specifically, he computes
  $${F}_{i}^{*}=(g^{a_{t-1}})^{Sr_{i}^{t-1}}{\cdots} (g^{a_{1}})^{Sr_{i}}(g^{s_{core}}) $$
  $${h}_{i}^{*}=(g^{b_{2t-2}})^{Sr_{i}^{2t-2}}{\cdots} (g^{b_{1}})^{Sr_{i}} $$
  If ${F}_{i}^{*}=g^{F_{i}}$ and ${h}_{i}^{*}=g^{h_{i}}$, then *S**r*_*i*_ accepts *F*_*i*_ and *h*_*i*_, otherwise he rejects.
- **Publish.** *D* computes
  $$s_{i}=d_{i}-s_{core} $$
  for 1 ≤ *i* ≤ *m*. Then *D* publishes *s*_1_, *s*_2_, ⋯ , *s*_*m*_ that can be seen by anyone including the servers. However, only the servers can use *s*_1_, *s*_2_, ⋯ , *s*_*m*_ to generate answer shares that can be used by the querier to recover the corresponding needed result.
- **Query.** The Querier *Q* sends servers a query that he wants to know a *data* that can be described as the following equation:
  2 $$ \mathit{data}{\kern-.5pt}={\kern-.5pt}d_{i_{1}}d_{i_{2}}{\kern-.5pt}+d_{i_{3}}d_{i_{4}}{\kern-.5pt}+\cdots+d_{i_{k_{1}-1}}d_{i_{k_{1}}}{\kern-.5pt}+d_{i_{k_{1}+ 1}}{\kern-.5pt}+\cdots++d_{i_{k_{1}+k_{2}}}{\kern-.5pt}, $$
  where $1\leq i_{1},i_{2},\cdots ,i_{k_{1}+k_{2}}\leq m$.
- **Answer.** If the *i*-th server *S**r*_*i*_ wishes to answer *Q*, then he will generate a *S**h**a**r**e*_*i*_ by calculating with *s*_1_, *s*_2_, ⋯ , *s*_*m*_, *F*_*i*_ and *h*_*i*_ as follows:
  $$\begin{array}{@{}rcl@{}} \mathit{Share}_{i} \!&=&\! (F_{i}+s_{i_{1}})(F_{i}+s_{i_{2}})+\cdots+(F_{i}+s_{i_{k_{1}-1}})(F_{i}+s_{i_{k_{1}}})\\ &&+\, (F_{i}+s_{i_{k_{1}+ 1}})+\cdots+(F_{i}+s_{i_{k_{1}+k_{2}}})-\frac{k}{2}h_{i} \end{array} $$
  After that, *S**r*_*i*_ encrypts *S**h**a**r**e*_*i*_ as $C_{i}^{share}=Enc_{pk_{Q}}(Share_{i})$ with *Q*’s public key. Then, *S**r*_*i*_ sends $C_{i}^{share}$ to *Q*.
- **Recover.** If *Q* collects at least *t* correct and different shares, he can recover the *data* as described in Eq. 2. Without loss of generality, we assume the *t* shares come from *S**r*_1_, *S**r*_2_, ⋯ , *S**r*_*t*_. First, *Q* should verify the validations of *S**h**a**r**e*_1_, *S**h**a**r**e*_2_ and *S**h**a**r**e*_*t*_. Specifically, *i* from 1 to *t*, *Q* computes
  $$g^{F_{i}}=(g^{a_{t-1}})^{Sr_{i}^{t-1}}{\cdots} (g^{a_{1}})^{Sr_{i}}(g^{a_{0}})=g^{a_{t-1}Sr_{i}^{t-1}+\cdots+a_{1}Sr_{i}+s_{core}} $$
  $$g^{h_{i}}=(g^{b_{2t-2}})^{Sr_{i}^{2t-2}}{\cdots} (g^{b_{1}})^{Sr_{i}}=g^{b_{2t-2}Sr_{i}^{2t-2}+\cdots+b_{1}Sr_{i}} $$
  After that, with $s_{i_{1}},s_{i_{2}},\cdots ,s_{i_{m}}$ and bilinear map *e*, *Q* further computes
  $$\begin{array}{@{}rcl@{}} {E_{i}^{1}}&=&e(g^{F_{i}}g^{s_{i_{1}}},g^{F_{i}}g^{s_{i_{2}}})e(g^{F_{i}}g^{s_{i_{3}}},g^{F_{i}}g^{s_{i_{4}}})\\ && {\cdots} e(g^{F_{i}}g^{s_{i_{k_{1}-1}}},g^{F_{i}}g^{s_{i_{k_{1}}}})\\ {E_{i}^{2}}&=&e(g_{i}^{F_{i}}g^{s_{i_{k_{1}+ 1}}}g_{i}^{F_{i}}g^{s_{i_{k_{1}+ 2}}}{\cdots} g_{i}^{F_{i}}g^{s_{i_{k_{1}+k_{2}}}},g)\\ \end{array} $$
  If
  $${E_{i}^{1}}{E_{i}^{2}}/e(g^{h_{i}},g^{\frac{k}{2}})=e(g^{\mathit{Share}_{i}},g), $$
  then *Q* considers that *S**h**a**r**e*_*i*_ is correctly computed by *S**r*_*i*_. If all *S**h**a**r**e*_1_, *S**h**a**r**e*_2_ and *S**h**a**r**e*_*t*_ are correctly computed by senders, then *Q* can recover the *data* with *S**h**a**r**e*_1_, *S**h**a**r**e*_2_ and *S**h**a**r**e*_*t*_. Specifically, *Q* can reconstruct a polynomial of degree *t* − 1 by *Lagrange interpolating* as follow:
  $$\widetilde{F}(x) = \sum\limits_{i = 1}^{t} \mathit{Share}_{i} \prod\limits_{j = 1,j\neq i}^{t}\frac{Sr_{j}-x}{Sr_{j}-Sr_{i}} $$
  Finally, $\widetilde {F}(0)$ equals to *data*.
